# Deep-learning prediction of cardiovascular outcomes from routine retinal images in individuals with type 2 diabetes

**DOI:** 10.1186/s12933-024-02564-w

**Published:** 2025-01-02

**Authors:** Mohammad Ghouse Syed, Emanuele Trucco, Muthu R. K. Mookiah, Chim C. Lang, Rory J. McCrimmon, Colin N. A. Palmer, Ewan R. Pearson, Alex S. F. Doney, Ify R. Mordi

**Affiliations:** 1 VAMPIRE project, Computing, School of Science and Engineering, University of Dundee, Dundee, USA; 2 Division of Cardiovascular Research, School of Medicine, University of Dundee, Dundee, DD1 9SY UK; 3 Division of Systems Medicine, School of Medicine, University of Dundee, Dundee, UK; 4 Division of Population Health and Genomics, School of Medicine, University of Dundee, Dundee, UK; 5 Tuanku Muhriz Royal Chair, National University of Malaysia, Bangi, Malaysia

**Keywords:** Artificial intelligence, Retina, Cardiovascular risk, Diabetes

## Abstract

**Background:**

Prior studies have demonstrated an association between retinal vascular features and cardiovascular disease (CVD), however most studies have only evaluated a few simple parameters at a time. Our aim was to determine whether a deep-learning artificial intelligence (AI) model could be used to predict CVD outcomes from routinely obtained diabetic retinal screening photographs and to compare its performance to a traditional clinical CVD risk score.

**Methods:**

We included 6127 individuals with type 2 diabetes without myocardial infarction or stroke prior to study entry. The cohort was divided into training (70%), validation (10%) and testing (20%) cohorts. Clinical 10-year CVD risk was calculated using the pooled cohort equation (PCE) risk score. A polygenic risk score (PRS) for coronary heart disease was also obtained. Retinal images were analysed using an EfficientNet-B2 network to predict 10-year CVD risk. The primary outcome was time to first major adverse CV event (MACE) including CV death, myocardial infarction or stroke.

**Results:**

1241 individuals were included in the test cohort (mean PCE 10-year CVD risk 35%). There was a strong correlation between retinal predicted CVD risk and the PCE risk score (*r* = 0.66) but not the polygenic risk score (*r* = 0.05). There were 288 MACE events. Higher retina-predicted risk was significantly associated with increased 10-year risk of MACE (HR 1.05 per 1% increase; 95% CI 1.04–1.06, *p* < 0.001) and remained so after adjustment for the PCE and polygenic risk score (HR 1.03; 95% CI 1.02–1.04, *p* < 0.001). The retinal risk score had similar performance to the PCE (both AUC 0.697) and when combined with the PCE and polygenic risk score had significantly improved performance compared to the PCE alone (AUC 0.728). An increase in retinal-predicted risk within 3 years was associated with subsequent increased MACE likelihood.

**Conclusions:**

A deep-learning AI model could accurately predict MACE from routine retinal screening photographs with a comparable performance to traditional clinical risk assessment in a diabetic cohort. Combining the AI-derived retinal risk prediction with a coronary heart disease polygenic risk score improved risk prediction. AI retinal assessment might allow a one-stop CVD risk assessment at routine retinal screening.

**Graphical abstract:**

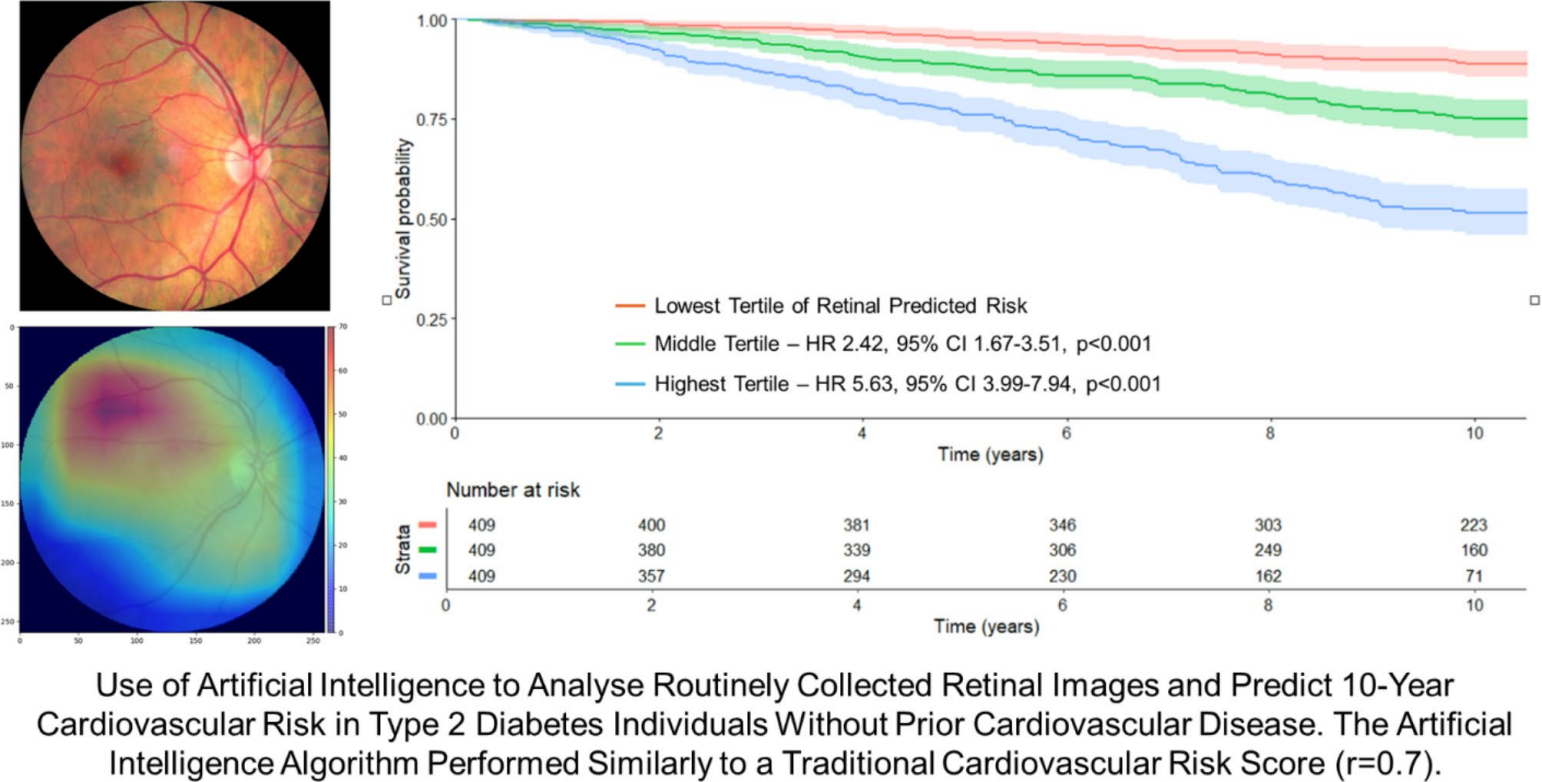

## Introduction

Cardiovascular disease (CVD) is the leading cause of morbidity and mortality among individuals with type 2 diabetes mellitus [1]. Diabetes is associated with accelerated incidence and progression of CVD [2] and CVD prevention plays a key role in diabetes management [3]. Recent trials have demonstrated that sodium-glucose co-transporter 2 inhibitors (SGLT2i) [4] and glucagon-like peptide 1 receptor agonists (GLP1-RA) [5] reduce the incidence of major cardiovascular events in individuals with type 2 diabetes at increased CVD risk, hence opportunities for improved CVD risk prediction in these patients might help to select patients who would derive particular benefit from these treatments.

In many healthcare systems, including in the United Kingdom, individuals with diabetes undergo routine retinal screening to identify the presence of diabetic retinopathy or maculopathy [6]. Such screening photographs allow direct visualization of the retinal microvasculature that could be used to inform on CVD risk. Several studies have identified the presence of retinopathy as an independent CVD risk predictor [7–10]. Subsequent studies have found associations between quantitative retinal vascular parameters such as vessel diameter, tortuosity and fractal dimension and increased CVD risk [11–16]. Measurement of these parameters can however be time-consuming and is not routinely performed in clinical practice.

Retinal photographs are ideal for analysis by artificial intelligence (AI) methodology [17]. Use of AI can not only reduce analysis time, but also provides the opportunity to take full advantage of the data contained within the image as opposed to just one or two parameters. Recent work has identified the ability of AI to predict cardiovascular risk factors and outcomes from retinal images with high diagnostic accuracy [18–21]. There have been few cohort studies reporting the feasibility of using AI to determine an individual’s CVD risk from a retinal photograph. The retinal microvasculature, directly observable with comparatively inexpensive devices, might not only provide an assessment of environmental risk factors associated with CVD, but it may also be reflective of an individual’s genetic susceptibility to these factors. The concept of using a retinal photo, perhaps combined with a marker of genetic CVD risk, as a one-stop assessment of global CVD risk is certainly appealing [11]. Little work exists on whether retinal images from multiple visits could be used to provide a dynamic assessment of CVD risk.

The aim of this study was to use a deep-learning AI model to predict the incidence of major adverse cardiovascular events (MACE) from routinely obtained diabetes retinal screening (DRS) photographs in individuals without prior myocardial infarction or stroke, determine the model’s prognostic performance when combined with genetic risk scores and ascertain whether changes in retinal AI-predicted CVD risk could be used to predict outcome.

## Methods

### Study cohort

We evaluated patients from the Genetics of Diabetes Audit and Research in Tayside Scotland (GoDARTS) study. Full cohort details have been previously described [22]. GoDARTS is a case-control cohort study conducted in Tayside, Scotland and includes 10,149 individuals with type 2 diabetes and 8,157 individuals without type 2 diabetes at the time of recruitment. A comprehensive set of baseline data were obtained at recruitment including clinical and lifestyle parameters, and consent was given for electronic health record linkage for past and future clinical events and healthcare contacts such as prescribing, laboratory tests, eye screening, hospitalisations and deaths. A blood sample was obtained at the time of recruitment for genotyping, with genome-wide association performed using various genotyping arrays as previously described. The GoDARTS study and electronic health record (EHR) linkage has been approved by the East of Scotland Research Ethics Committee. The EHR is fully anonymized and provided to researchers through robust information governance protocols administered by the Health Informatics Centre (HIC) Trusted Research Environment (TRE), including overarching research ethics approval for studies conducted within the TRE.

We studied patients with type 2 diabetes with no history of myocardial infarction (MI) or stroke (ICD codes I21-I23 and I60-I63) prior to the date of the first available retinal photograph (which was taken as the study entry date for this analysis). This date was also used to determine baseline demographic and clinical characteristics in order to calculate clinical 10-year cardiovascular risk using the Pooled Cohort Equation [23]. Clinical variables nearest the study entry date were used. Where there was more than one measure of a variable (e.g. cholesterol, blood pressure) we used the median over the 3 years prior to study entry. A genome wide polygenic risk score (PRS) for coronary heart disease (CHD) was constructed from the genome-wide genotyping data available in the GoDARTS bioresource using published data as described previously by Khera et al. [11, 24]. This PRS was taken from a GWAS involving over 180,000 individuals and included over 6.6 million genetic variants. The PRS was z-standardised.

### Retinal imaging

Retinal photography was captured during routine annual diabetic retinal screening. The Scottish Diabetic Retinal Screening programme uses standardized protocols (details available at https://www.ndrs.scot.nhs.uk/). In brief, all the retinal images have 45° field of view and are macula-centred. All photographs had been previously assessed by a trained ophthalmologist for the presence of retinopathy or maculopathy for clinical purposes at the time of photography. We used the earliest available retinal photograph to train and test the DL model, with images from both eyes used.

### Image pre-processing and data split

**Fig. 1:**
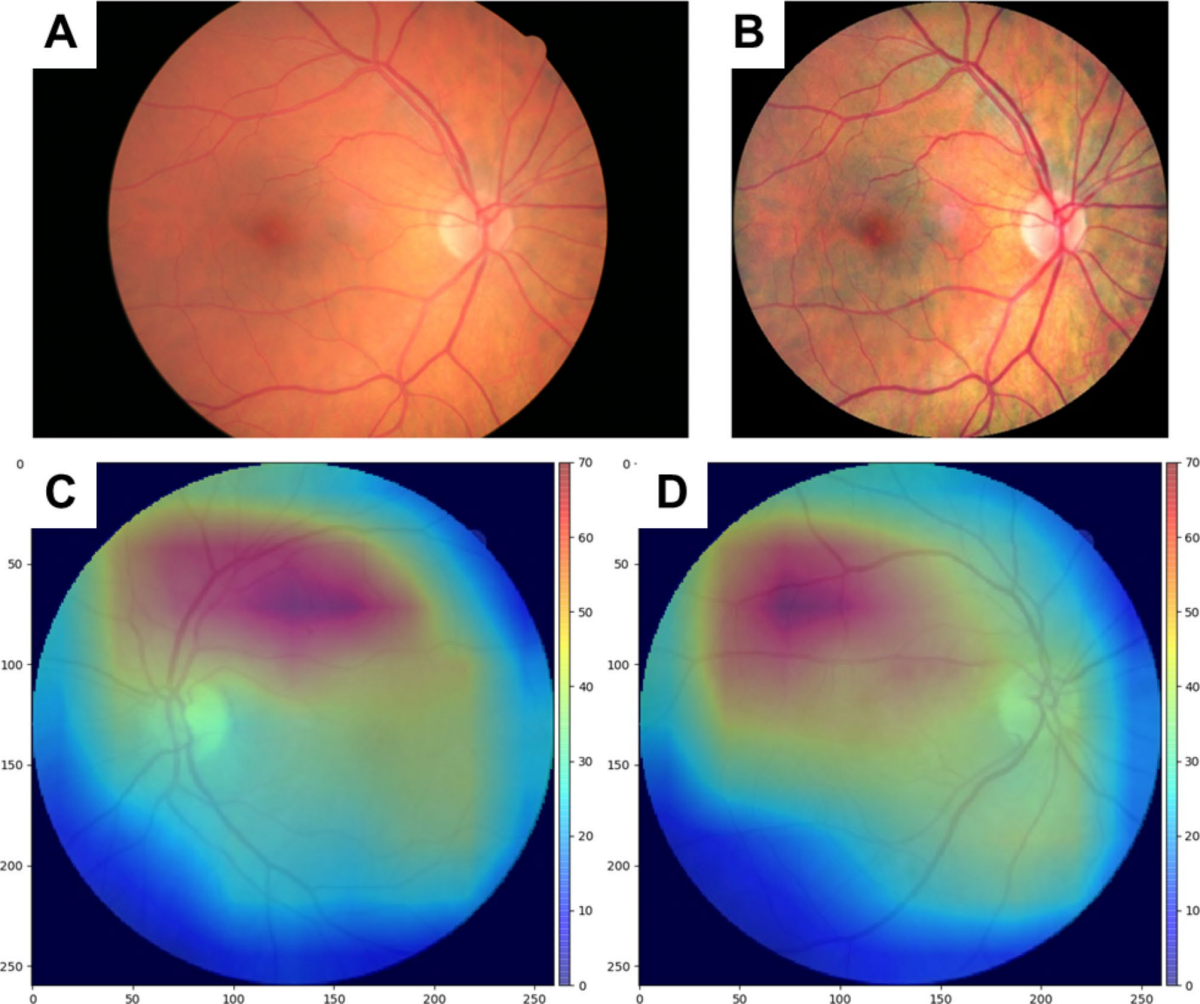
Example of pre-processing result (top) and Average of Grad-CAM heatmaps for PCE risk score prediction superimposed on a sample left eye and right eye retina (bottom). **A** Original retinal image; **B** pre-processed image; **C** heatmap for left eye retinas; **D** heatmap for right eye retinas

The DRS images used had 13 different image sizes, three of which formed the vast majority (98.9%) of the data: 2336 × 3504 pixels (92.7%), 2304 × 3456 (4%) and 1696 × 2544 (2.2%). Smaller dimensions accounted for only 1% of the images. There were also significant variations in these images in terms of luminosity, color, focus and quality. To reduce the effects of these variations in training a DL model we pre-processed the images in three steps as described previously [25, 26]. First, we fit a circle on the original retinal image to remove the non-retinal (black background) region. Second, we resized the image to a standard size (260 × 260) and performed contrast limited adaptive histogram equalization (CLAHE) on R, G, and B color channels separately. Finally, pixel intensities were normalized to [0, 1]. A sample retinal image before and after pre-processing is shown in Fig. 1A and B.

The cohort was randomly split into three groups: 70% training, 10% validation and 20% testing. To avoid information leak, all retinal images belonging to same individual were allocated to one group only.

### Deep learning architecture and training

A deep convolutional neural network (CNN) was used to predict risk of MACE from the retinal images. We adopted EfficientNet-B2 [27], part of the EfficientNet family of architectures, a well-tested model that has been reported to achieve excellent performance in image classification tasks [28]. The EfficientNet-B2 architecture was modified by replacing its fully connected layer with a global average pooling layer followed by a single output node with sigmoid activation. The convolutional layers were unchanged. We used 260 × 260 input images following recommendations for best performance suggested by the authors of the EfficientNet-B2 [27]. As transfer learning, we initialized the weights of all the convolutional layers with pre-trained weights on ImageNet [28], allowing us to avoid the expensive task of training the network from scratch [29]. The model contained a total of 7.7 M trainable parameters and all of these were trained.

The training split was used to train the model parameters, with the validation split used to avoid overfitting of the model on the trained dataset. Binary cross-entropy was used as the loss function. We employed the Nadam optimizer from Keras (https://keras.io/api/optimizers/Nadam/) with an initial learning rate of 0.001, reduced by a factor of 0.1 if the validation loss did not improve within 5 consecutive epochs (https://keras.io/api/callbacks/reduce_lr_on_plateau/). The minimum learning rate was set to 10^− 5^. The model was trained with a batch size 32 for 50 epochs, a number we empirically observed to be sufficient for convergence due to transfer learning. A random horizontal flip and random rotation were used for image augmentation of the training images. During validation, early stopping (https://keras.io/api/callbacks/early_stopping/) was implemented, halting training if there was no improvement in validation loss for 20 consecutive epochs. The weights set with best validation performance were saved for the final testing phase.

All work was carried out in the University of Dundee HIC Trusted Research Environment [30] on a NVIDIA TITAN Xp GPU. Python 3.6 was used for code development with libraries opencv [31], scikit-learn [32] for image processing, and Keras 2.2.2[33] with tensorflow 1.9.0 as back-end for building [34], training and testing DL models.

### Heatmap visualization

We used gradient-based class activation mapping (grad-CAM) [35] to visualize the regions in the input images that contained critical information for MACE prediction. We applied grad-CAM to the last convolutional layer of the modified EfficientNet-B2.

### Prediction of 10-year MACE

The ML model was used to calculate the predicted 10-year MACE (predMACE_10_) from the date of the retinal image in the test data. Since MACE events are linked to each individual (rather than specifically to the left or right eye), we included only individuals with retinal images from both eyes in the test dataset. Following previous work that demonstrated improved predictive accuracy by averaging results from multiple left and right eye retinal images of the same individual [36], we calculated an individual-level MACE prediction by averaging the predMACE10 scores from both the left and right eye images.

As well as prediction of MACE from baseline retinal images we also performed a longitudinal analysis to evaluate changes in retinal predicted MACE risk over time. We evaluated retinal images from all individuals in the test dataset who had a further retinal photograph available for analysis within 3 years of the baseline image. We calculated the difference between the predMACE_10_ score from the baseline retinal image to the subsequent image. To account for the second image being at different timepoints within the 3-year window we calculated the rate of change as follows:

$$ \begin{gathered} \quad Predicted~MACE_{{10}} ~difference \hfill \\ = ~\frac{{predMACE_{{10}} ~from~last~available~image - predMACE_{{10}} ~from~first~available~image}}{{Duration~between~last~and~first~image~capture~dates}} \hfill \\ \end{gathered} $$

Thus positive values suggested an increased MACE risk, while negative values indicated a reduced MACE risk.

### Clinical outcomes

Clinical events from the date of study entry to December 2018 were obtained from healthcare electronic medical records from the GoDARTS cohort as previously described [11]. The primary outcome for this analysis was time to first MACE, defined as cardiovascular death, non-fatal myocardial infarction or non-fatal stroke using ICD codes. Causes of death were obtained from the General Register of Scotland, with any ICD-10 code from I00-I99 within the first two causes of death being classified as a cardiovascular death. Follow-up was limited to 10 years to correspond to the duration of risk prediction from the PCE risk score.

### Statistical analysis

Continuous variables are reported here as mean ± standard deviation or median and interquartile range as appropriate and categorical variables as number and percentage.

Following recent common practice [18, 36–38], we used the area under receiver operating characteristic curve (AUC; ROC) for 10-year MACE as our primary evaluation to analyse the model’s performance on test data, defined in detail elsewhere [25]. To assess the statistical significance of the model performance, non-parametric bootstrap sampling was used on test data. We used 2000 random samples with replacement from the test data, where each sample size was the same as that of the test data. We computed evaluation metrics from each bootstrap sample and 95% confidence intervals (CI) was reported from the distribution obtained from all bootstrap samples.

Associations between predMACE_10_, PCE and PRS were assessed using Pearson’s correlation coefficient. To evaluate the association between the predMACE_10_ and clinical outcomes, we used Cox proportional hazard regression analysis and the Kaplan-Meier (KM) estimator used on tertiles of predMACE_10_ from the test data. To quantify the association further, we performed with multivariable adjustment for clinical and genetic cardiovascular risk calculated by the PCE and the coronary heart disease polygenic risk score.

To determine the comparative prognostic performance of predMACE_10_ we calculated the area under the curve for the association with MACE at 10 years. To determine the association between the predMACE_10_ change and clinical outcomes, the KM estimator was used on the top 20% and the bottom 80% of the computed predMACE_10_ difference from the test data, with Cox regression used to quantify the association with 10-year MACE.

Survival analysis was carried out using R (3.2.5) with ‘survival’ package and ‘survminer’ package (for the plots). All tests were two-sided and a p value < 0.05 considered significant.

## Results

### Baseline characteristics

In total, of the 10,149 individuals with type 2 diabetes in GoDARTS we included 6,127 individuals who had available retinal photographs (total 12,859 retinal photographs), divided into ~ 70% training (4281 individuals with 8990 images), ~ 10% validation (605 individuals with 1271 images) and ~ 20% testing (1241 individuals with 2598 images). Full baseline characteristics are described in Table 1. There were no significant differences in the data distribution between the training, validation and test cohorts. In the overall cohort, the mean age at baseline was 67 ± 11 years, with 55% of the cohort being male. As would be expected from a diabetes cohort, clinical cardiovascular risk was high, with a mean PCE 10-year cardiovascular disease risk score of 34 ± 20%.

**Table 1:** Baseline characteristics of whole cohort and data splits

Feature	Overall	Train	Validation	Test
Number of participants	6127	4281	605	1241
Total number of Images Used	12,859	8990	1271	2598
Right eye images (%)	5869 (45.6)	4104 (45.7)	577 (45.4)	1188 (45.7)
Age at imaging (years)	67 (11)	67 (11)	67 (11)	68 (12)
Male (%)	3368 (55.0)	2350 (54.9)	317 (52.4)	701 (56.4)
Smoking history (%)	3346 (50.4)	2367 (50.9)	341 (51.4)	638 (48.0)
Glycated haemoglobin (%)	7.5 (1.1)	7.5 (1.1)	7.6 (1.2)	7.6 (1.2)
Median duration of diabetes (years)	7 (4, 12)	7 (4, 12)	7 (4, 12)	7 (4, 12)
Systolic blood pressure (mmHg)	139 (11)	139 (11)	139 (11)	140 (12)
Diastolic blood pressure (mmHg)	76 (8)	76 (8)	76 (8)	76 (8)
Body mass index (kg/m2)	31 (6)	31 (6)	31 (6)	31 (6)
Total cholesterol (mmol/l)	4.4 (0.9)	4.4 (0.9)	4.4 (0.8)	4.4 (0.8)
HDL cholesterol (mmol/l)	1.3 (0.4)	1.4 (0.4)	1.3 (0.4)	1.3 (0.3)
PCE risk score (%)	34 (20)	33 (20)	34 (21)	35 (20)
Z-standardised polygenic risk score	6.92 (0.60)	6.93 (0.60)	6.90 (0.62)	6.91 (0.62)

Continuous variables reported as mean (std, IQR) unless otherwise stated, categorical as number (percentage). std - standard deviation; IQR - inter quartile range; HDL- high-density lipoprotein; PCE—pooled cohort equation

### Cardiovascular disease risk prediction using deep learning

For training, validation and testing the EfficientNet-B2 model, retinal images of the left and right eyes at baseline (earliest available image) were considered.

There was no significant difference in model performance between males and females. For prediction of 10-year MACE the model achieved an AUC of 0.691 (95% CI 0.666–0.715) on the complete test dataset; 0.688 (0.657–0.719) using left eye images only; 0.689 (0.660–0.718) using right eye images only and 0.697 (0.663–0.731) using the mean of both eyes. Given the similar performance using all four methods we used the individual level (i.e. the mean of both eyes for each individual) predMACE_10_ for further analyses.

The grad-CAM heatmaps indicated that the optic disc, macula and vasculature were the most important retinal features for predicting 10-year MACE. Figure 1C and D show average of grad-CAM heatmaps computed for all left eye and right eye retinal images for CVD risk prediction in the test dataset, superimposed on a sample retinal image to illustrate the anatomical correspondence for highlighted areas.

### Association between retinal predicted MACE, clinical CVD risk and CHD PRS

**Fig. 2:**
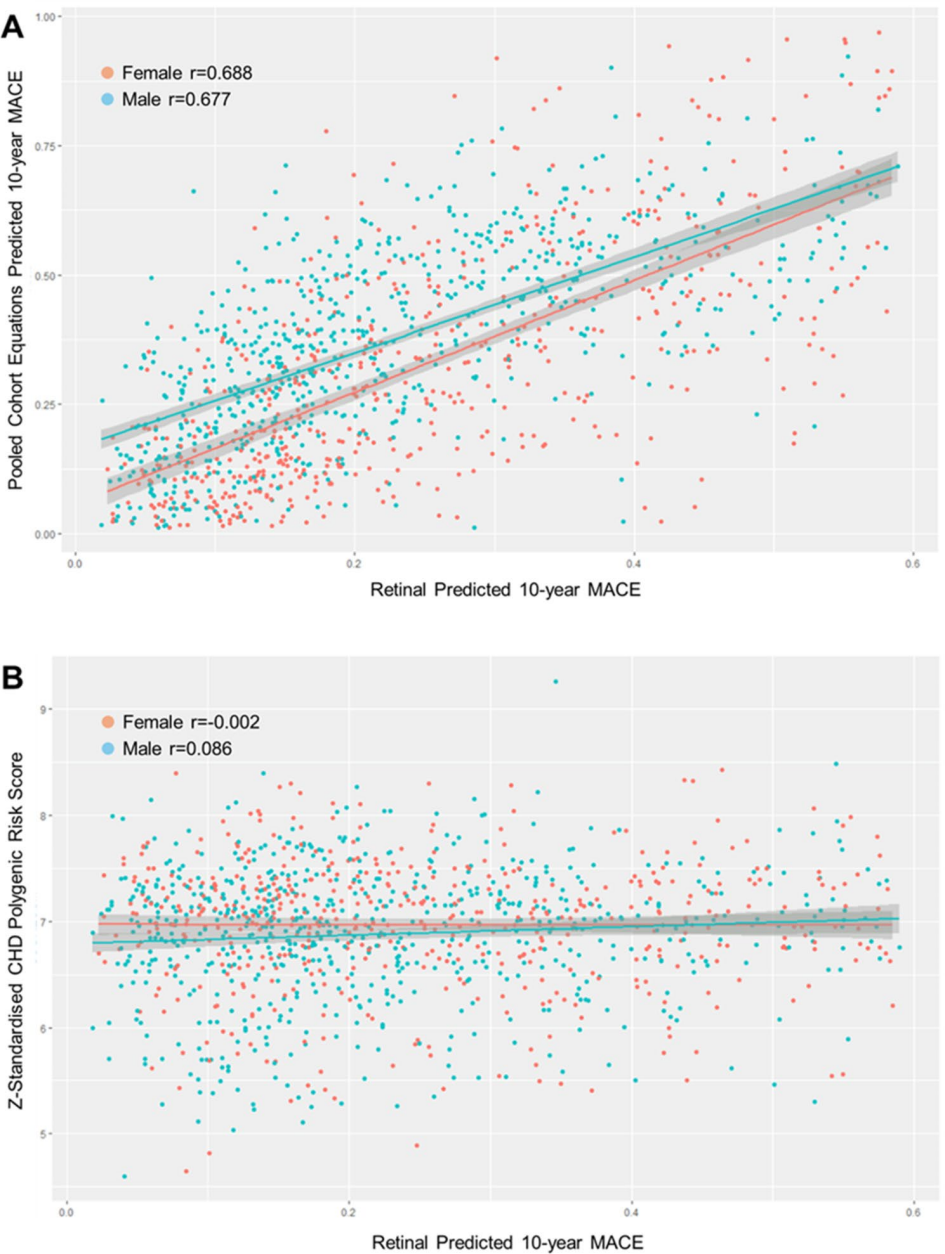
Correlations between individual-level retinal predicted 10-year MACE, clinical CVD risk and genetic CHD risk. **A** Predicted 10-year MACE vs. CHD PRS. **B** Predicted 10-year MACE vs. PCE risk score. Red dots represent females and blue dots represent males

Retinal predMACE_10_ appeared to correlate predominantly with clinical rather than genetic CVD risk. There was a strong correlation between retinal predMACE_10_ and actual PCE CVD risk (*r* = 0.66). There was no correlation between retinal predMACE_10_ and the coronary heart disease PRS (*r* = 0.05). Similarly, there was little correlation between the PRS and the actual PCE CVD risk (*r*=-0.02) (Fig. 2).

### Retinal predicted MACE and clinical outcome

**Fig. 3:**
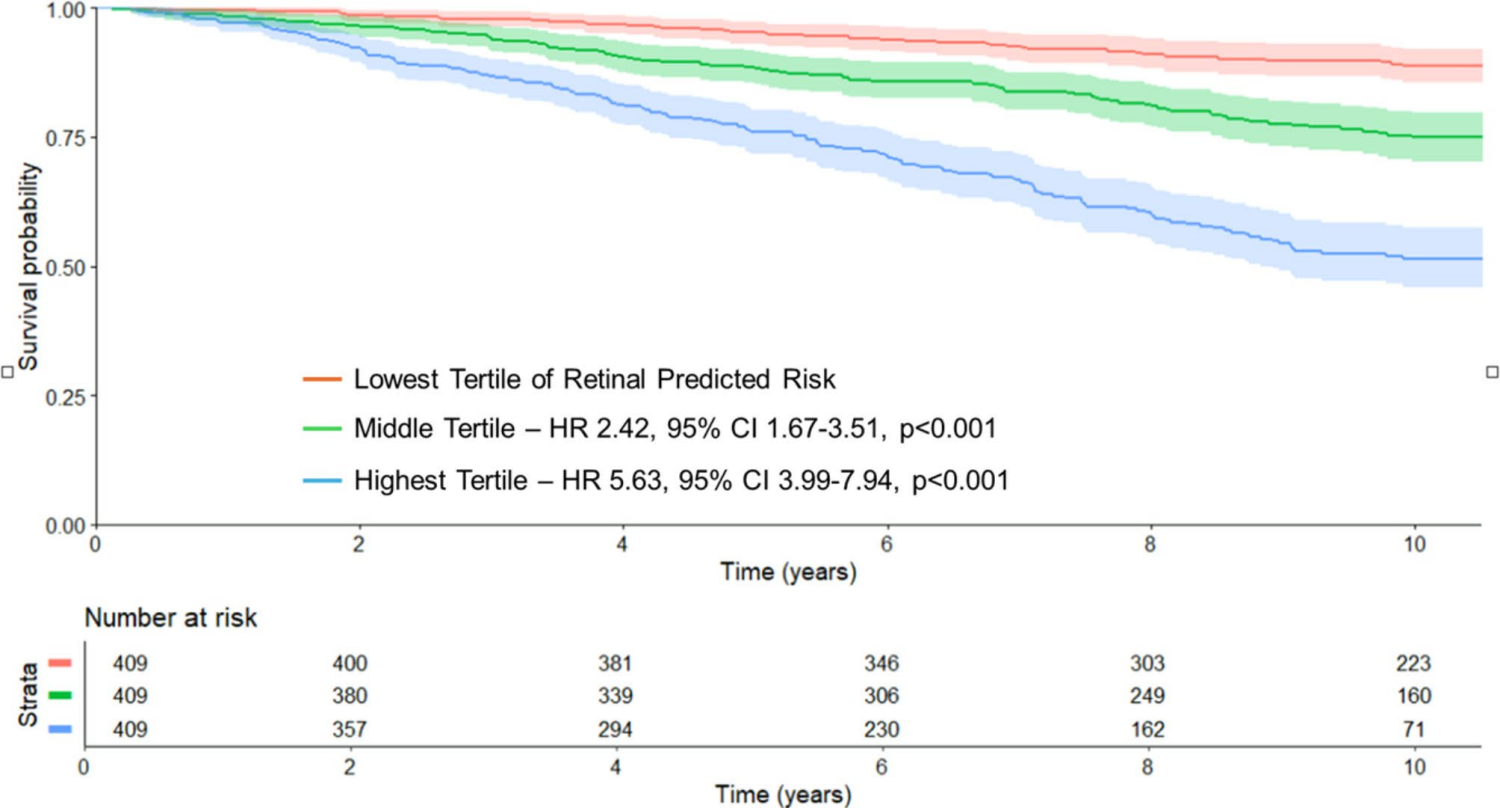
Major adverse cardiovascular events stratified by retinal predicted CVD risk. Kaplan-Meier curves showing MACE-free survival at 10 years stratified by tertiles of retinal predicted CVD risk derived from individual-level prediction at baseline

In the test cohort, over a median follow-up duration of 9.1 years, there were 288 MACE events, including 244 CV deaths, 71 non-fatal MIs and 50 non-fatal strokes within 10 years from the date of retinal imaging. Higher retinal predMACE_10_ was significantly associated with increased 10-year risk of MACE (HR 1.05 per 1% increase; 95% CI 1.04–1.06, *p* < 0.001). Individuals in the highest tertile of retinal predMACE_10_ (predicted 10-year MACE 28–59%) had a significantly higher risk of MACE than those in the lower (2–15%) and middle (15–28%) tertiles (compared to lowest tertile - middle tertile HR 2.42; 95% CI 1.67–3.51, *p* < 0.001; highest tertile HR 5.63; 95% CI 3.99–7.94, *p* < 0.001) (Fig. 3).

### Incremental prognostic value of retinal PredMACE10 in addition to PCE risk score and CHD PRS

After adjustment for the CHD polygenic risk score, higher retinal predMACE_10_ remained significantly associated with increased 10-year risk of MACE incidence (HR per 1% increase 1.05; 95% CI 1.04–1.06, *p* < 0.001). Similarly, higher retinal predMACE_10_ remained significantly associated with higher risk of 10-year MACE (HR per 1% increase 1.03; 95% CI 1.02–1.04, *p* < 0.001) after adjustment for the PCE risk score. In a model incorporating both the CHD polygenic risk score and the PCE risk score retinal predMACE_10_ remained independently associated with MACE at 10 years (HR per 1% increase 1.03; 95% CI 1.02–1.04, *p* < 0.001).

The retinal predMACE_10_ score demonstrated similar performance to the PCE risk score for prediction of MACE (AUC—retinal predMACE_10_ 0.697 (95% CI 0.663–0.731); PCE 0.697 (95% CI 0.663–0.730) and better performance than the CHD polygenic risk score alone (AUC 0.575; 95% CI 0.538–0.613). There was a small but significant improvement in risk prediction when the PCE risk and CHD polygenic risk scores were added to the retinal predMACE_10_ score (0.728; 95% CI 0.695–0.761).

### Longitudinal changes in retinal predicted MACE and subsequent clinical outcomes

1,072 individuals in the test cohort had an available second retinal photograph within 3 years of the baseline image. In this group, there were 226 MACE events after the second image. The median change in retinal predMACE_10_ was + 2.4% (IQR − 0.6–5.4%).

**Fig. 4:**
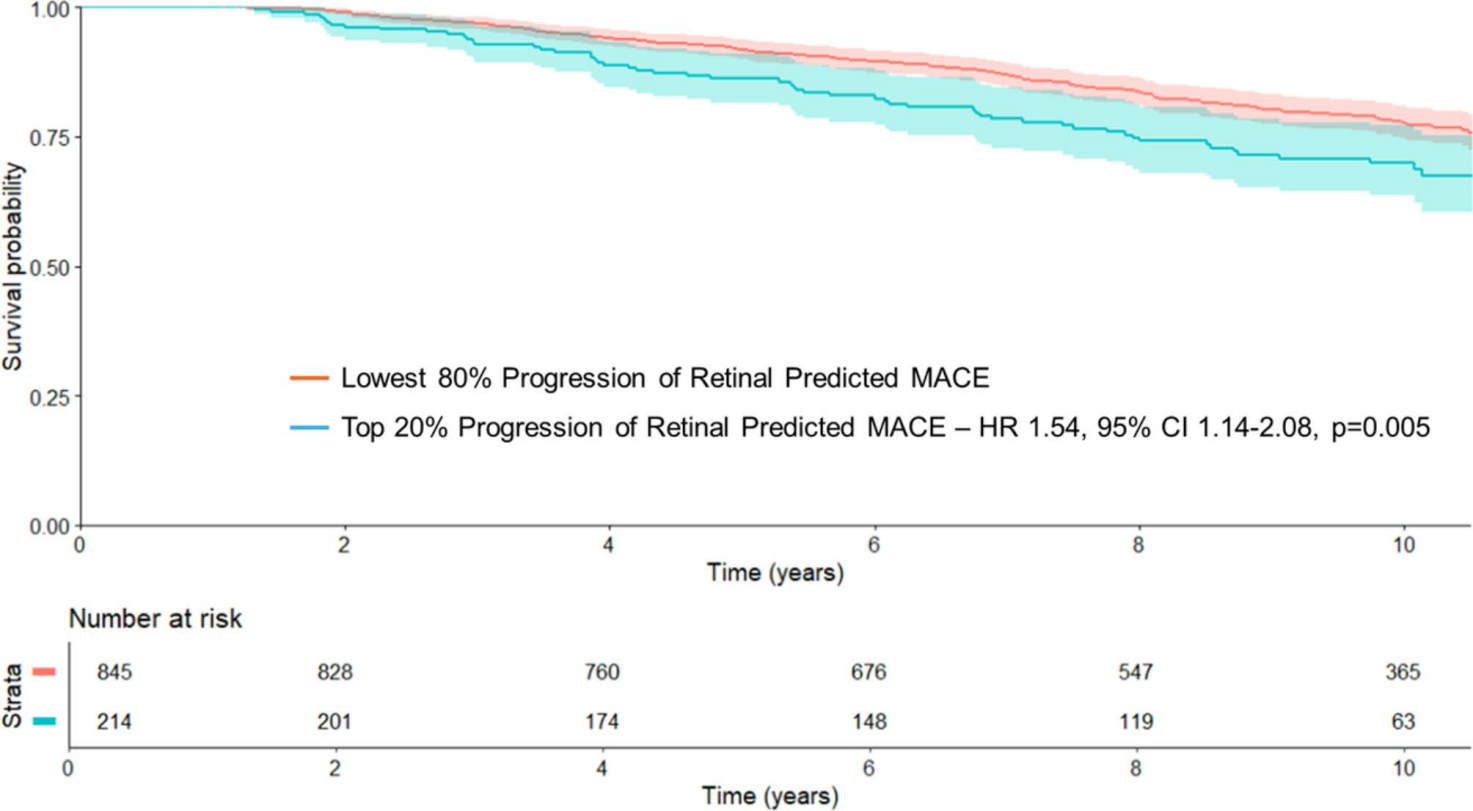
Major Adverse Cardiovascular Events Based on Progression of Retinal Predicted CVD Risk

Individuals in the top quintile of predMACE_10_ difference (i.e. those with the largest increase in predMACE_10_ between the first and second retinal images, ranging from an increase of 3.2–37% per year) had a significantly higher risk of incident MACE (HR 1.54, 95% CI 1.14–2.08, *p* = 0.005) compared to the rest of the cohort (Fig. 4).

Kaplan-Meier curves showing MACE-free survival in individuals who had a second retinal photograph, comparing individuals with most accelerated progression of retinal predicted CVD risk (top 20%) and those with slower progression or reduced retinal predicted CVD risk (bottom 80%).

## Discussion

We have identified a number of key findings. First, we have shown that it is feasible to use a deep learning approach to predict CVD risk in individuals with type 2 diabetes without CVD at baseline from routine clinical retinal photographs. Second, we have shown that this retinal CVD risk prediction model (predMACE_10_) performs similarly to a traditional clinical risk score and is able to predict major adverse cardiovascular events independent of both traditional clinical and genomic CVD risk. Third, we have shown that the addition of retinal predMACE_10_ to genomic risk provides incremental prognostic value for prediction of MACE compared to a traditional clinical risk score. Finally, we have shown that increases in retinal predMACE_10_ are also associated with increased risk of future MACE, raising the possibility that this model could be used in routine annual diabetic eye screening.

There has been significant research interest in using deep-learning approaches to predict CVD risk and outcomes from retinal photography [39]. Previous studies have shown that AI can be used to predict CVD risk factors and outcomes such as mortality with high diagnostic accuracy [18–20]. This concept is particularly attractive: if CVD risk could be ascertained from routine clinical retinal images, this could avoid the need for multiple healthcare attendances. Our work confirms, in line with other studies, that a deep learning methodology is able to predict CVD risk, but, to our best knowledge, ours is the first study to compare retinal CVD risk prediction and the Pooled Cohort Equation clinical ASCVD risk score, demonstrating similar performance. Since retinal photographs can be relatively easily obtained but require expertise to interpret [40], an AI approach could allow adoption of rapid retinal CVD risk assessment in routine clinical care, including primary care (optometrists), saving time on regular blood pressure and cholesterol monitoring (for example) and reducing congestion in secondary care [41]. 

It has long been recognised that there is a significant genetic component to CVD risk, however beyond a simple binary assessment of the presence of a family member with established CVD, most clinical risk scores do not take this into account. Over the past decade advances in genotyping have allowed development of whole-genome polygenic risk scores that can predict coronary heart disease and CVD events [24, 42, 43]. As we have previously shown [11], individual markers of CVD risk can be combined with a polygenic risk score to provide incremental prediction of CVD risk. AI systems could make retinal assessment less time-consuming and more reproducible, and in this study the combination of retinal imaging and a polygenic risk score had incremental prognostic value over a standard clinical risk score.

The reproducibility of AI could provide further benefit if used as a serial assessment of CVD risk. We have, for the first time, shown that particularly high-risk patients can be identified by tracking longitudinal progression of retinal predMACE_10_. This might allow clinical implementation as it provides a measure of CVD risk that could be used as a treatment target. Furthermore, our algorithm was able to predict CVD risk with similar accuracy regardless of whether a single eye photograph or both were used, and whether the left or right eye was used. Again, this could aid clinical applicability as up to 20% patients will not have optimal imaging [44]. 

Our study does have limitations. It predominantly included individuals aged between 60 and 75; further work is required to investigate performance in older and younger individuals. As we used clinically available electronic health record data not all measurements were taken at the same time as retinal photographs were performed. Furthermore, the second retinal imaging assessment was not performed at the same time in each individual as these were performed as per clinical need—further studies should explore a standardized protocol for retinal image follow-up. Although we used electronic health record data, because of the unique patient identifier used in the Scottish healthcare system we are able to follow all individuals who stay within Scotland for clinical outcomes and vital status throughout life. Image size was reduced to 260 × 260, as is common in deep learning systems due to limitation of computational resources; larger images might improve performance and future work could assess whether would provide additional prognostic value. Our cohort was predominantly Caucasian; so further work is required to ensure that this approach can be used in other ethnicities. Further work is also required to externally validate and compare our ML model to other available algorithms.

In conclusion, we have shown that a deep-learning artificial intelligence methodology is able to accurately predict CVD risk from routine diabetic retinopathy screening photographs with a comparable performance to traditional clinical risk assessment. Longitudinal assessment of retinal risk also predicted adverse CVD events. Implementation of this AI approach alongside retinal screening programmes might be an efficient method of CVD risk assessment. Combining the AI-derived retinal risk with a coronary heart disease polygenic risk score improved risk prediction and underlines the future potential of a one-stop CVD risk assessment derived from retinal screening. Future work should be performed to help translate our exploratory findings into routine clinical practice.

## Data Availability

Patient electronic healthcare data was collected with approval from NHS Tayside Health Board by the Health Informatics Centre, University of Dundee (HIC). It was de-identified and linked with required fields specifically for this study, however, is still considered sensitive data and cannot be made publicly available. Requests for access can be made to HIC with the appropriate approvals.

## Abbreviations

AI: Artificial intelligence
CLAHE: Contrast limited adaptive histogram equalization
CNN: Convolutional neural network
CVD: Cardiovascular disease
DL: Deep learning
DRS: Diabetes retinal screening
EHR: Electronic health records
GLP1: RA–glucagon–like peptide 1 receptor agonists
GoDARTS: Genetics of diabetes audit and research in Tayside Scotland
Grad: CAM–gradient–based class activation mapping
GWAS: Genome–wide association study
HIC: Health informatics centre
ICD: International classification of diseases
MACE: Major adverse cardiovascular events
MI: Myocardial infarction
PCE: Pooled cohort equations
predMACE_10_: Retinal 10–year MACE prediction
PRS: Polygenic risk score
SGLT2i: Sodium–glucose co–transporter 2 inhibitors
